# Muertes evitables en la niñez: un análisis por departamento y municipio en Colombia (2000-2018)

**DOI:** 10.26633/RPSP.2021.64

**Published:** 2021-06-11

**Authors:** Maylen Liseth Rojas-Botero, Yadira Eugenia Borrero Ramírez, Flor de María Cáceres-Manrique

**Affiliations:** 1 Facultad Nacional de Salud Pública, Universidad de Antioquia Medellín Colombia Facultad Nacional de Salud Pública, Universidad de Antioquia, Medellín, Colombia.; 2 Facultad de Salud, Universidad Industrial de Santander Colombia Facultad de Salud, Universidad Industrial de Santander, Colombia.

**Keywords:** Salud del niño, causas de muerte, Colombia, Child health, cause of death, Colombia, Saúde da criança, causas de morte, Colômbia

## Abstract

**Objetivo.:**

Determinar la proporción de muertes potencialmente evitables en niños menores de cinco años de Colombia, según departamento y municipio de residencia, en el período 2000-2018.

**Métodos.:**

Se llevó a cabo un estudio ecológico en múltiples grupos y longitudinal. Se analizaron 33 departamentos y 1 118 municipios en 19 años. Se clasificaron y diferenciaron las muertes evitables (tratables, prevenibles y mixtas) de las difícilmente evitables y se calculó la proporción respecto al total. Por último, se crearon conglomerados de municipios y departamentos, representados en mapas coropléticos.

**Resultados.:**

Entre 2000 y 2018 se registraron en Colombia 228 942 defunciones de niños menores de cinco años, 91,4% de las cuales eran evitables (68,2% tratables, 6,8% prevenibles y 16,5% mixtas) sin diferencias según el sexo. La proporción de evitabilidad pasó del 93,5% al 88,5%. Cesar fue el departamento con mayor proporción de muertes evitables (94,1%) en contraste con Santander, donde se observó la proporción menos alta (89,0%); entre tanto, a nivel municipal, en 99 municipios la totalidad de las defunciones fueron potencialmente evitables, mientras que en Palmar (Santander) se encontró la proporción más baja (33,3%).

**Conclusiones.:**

Nueve de cada 10 muertes de niños menores de cinco años ocurridas en Colombia entre 2000 y 2018 podrían haberse evitado, principalmente a través de la atención médica oportuna y de calidad, con importantes brechas entre los territorios subnacionales.

## Abstract (en)

**Objective.:**

To determine the proportion of potentially avoidable deaths in children under five in Colombia, by department and municipality of residence, during the period from 2000 through 2018.

**Methods.:**

A multi-group and longitudinal ecological study was conducted in 33 departments and 1 118 municipalities over a period of 19 years. The deaths were classified as probably unavoidable or avoidable; the latter were then identified as treatable, preventable, or mixed; and a proportion was calculated relative to the total. Finally, clusters were created by municipality and by department and depicted in coropleth maps.

**Results.:**

Between 2000 and 2018, Colombia reported 228 942 deaths of children under five, of which 91.4% were avoidable (68.2% treatable, 6.8% preventable, and 16.5% mixed) with no difference by sex. At the national level, the proportion of avoidability declined from 93.5% to 88.5% during this period. César was the department with the largest proportion of avoidable deaths (94.1%), compared with Santander, which had the smallest (89.0%). At the municipal level, all the deaths were potentially avoidable in 99 municipalities, whereas Palmar (Santander) had the least (33.3%).

**Conclusions.:**

Nine out of 10 of the deaths that occurred in children under five in Colombia during 2000-2018 could have been avoided, mainly through early and better quality medical care. Sizable differences were observed between subnational territories.

## RESUMO

**Objetivo.:**

Determinar a proporção de óbitos potencialmente evitáveis em crianças menores de cinco anos na Colômbia, segundo departamento e município de residência, no período de 2000-2018.

**Métodos.:**

Estudo ecológico longitudinal de múltiplas coortes que analisou dados de 33 departamentos e 1 118 municípios na Colômbia ao longo de 19 anos. Os óbitos foram classificados e diferenciados entre causas evitáveis (tratáveis, preveníveis ou mistas) e dificilmente evitáveis, e a proporção de óbitos foi calculada em relação ao total. Por último, foram criados conglomerados de municípios e departamentos, representados em mapas coropléticos.

**Resultados.:**

Entre 2000 e 2018, foram registrados 228 942 óbitos em menores de cinco anos na Colômbia, dos quais 91,4% por causas evitáveis (68,2% por causas tratáveis, 6,8% por causas preveníveis e 16,5% por causas mistas), sem diferença por sexo. A proporção de evitabilidade teve uma redução, de 93,5% a 88,5%. A maior proporção de óbitos por causas evitáveis (94,1%) foi observada no departamento de Cesar, e a menor (89,0%), em Santander. Em 99 municípios, todos os óbitos foram potencialmente evitáveis. A menor proporção de óbitos evitáveis (33,3%) foi observada no município de Palmar (departamento de Santander).

**Conclusões.:**

Nove em cada 10 óbitos de menores de cinco anos ocorridos na Colômbia entre 2000 e 2018 poderiam ter sido evitados, principalmente com atenção médica de qualidade e oportuna. Foi constatada uma grande disparidade entre os territórios subnacionais.

El siglo XX fue testigo de una disminución importante en la mortalidad infantil a nivel mundial. De acuerdo con el Fondo de las Naciones Unidas para la Infancia (UNICEF, por su sigla en inglés) la oportunidad de sobrevivir a la niñez ha aumentado de manera significativa; la mortalidad de niños menores de cinco años (MNM5) disminuyó más de la mitad entre 1990 y 2018, al pasar de 93 a 39 muertes por cada mil nacidos vivos (NV) (1). No obstante, el avance en las tendencias ha sido desigual entre territorios y entre subgrupos poblacionales específicos de acuerdo con su pertenencia de clase social o étnica, características demográficas y de sexo (2,3).

Se ha demostrado que la mortalidad en la niñez es un indicador sensible de la salud, de las condiciones de vida, del desarrollo y del bienestar de una población, con capacidad para evidenciar la articulación de determinantes individuales, familiares, ambientales, comunitarios y sociales (4,5). El mejoramiento de las condiciones materiales y simbólicas de vida, del sistema de salud, y del contexto socioeconómico y político pueden incidir en gran medida en la probabilidad de supervivencia, en el riesgo de morir por causas potencialmente evitables y en la disminución de brechas sociales en la MNM5.

Si bien Colombia ha logrado una reducción sustancial en la MNM5, persisten desigualdades e inequidades territoriales, de clase y étnicas. Entre 1990 y 2018, la tasa disminuyó en un 60,0% al pasar de 35 a 14 defunciones en niños menores de cinco años (NM5) por cada 1 000 NV (1). Esta trayectoria muestra el impacto de políticas y programas sociales en salud; sin embargo, en 2018 murieron en el país cerca de 9 000 niños antes de cumplir sus primeros cinco años (6), la mayoría de ellos por causas evitables a través de intervenciones bien conocidas y de bajo costo (7).

Las muertes evitables pueden entenderse como fallecimientos innecesariamente precoces, de modo que “si todo lo que se tenía que hacer se hubiera hecho, dichas defunciones se hubiesen retrasado o evitado” (8). Si bien la aplicación del concepto se remonta a principios del siglo XX (9), fue hasta mediados de los años 70 cuando Rutstein et al. propusieron un concepto como indicador de la oportunidad y calidad de la atención en salud, y crearon una lista de enfermedades o eventos que no deberían –o deberían hacerlo solo eventualmente– desencadenar en la muerte (8). En la literatura científica, existen diversas acepciones para la mortalidad evitable; asimismo, se han propuesto varios términos para su estudio: de esta manera se distinguen hoy en día las muertes tratables de las prevenibles. Una muerte se considera tratable cuando todas o la mayoría de las defunciones por esa causa podrían evitarse a través de la atención médica de buena calidad (Grupo A), por lo que las características y organización del sistema de salud son determinantes fundamentales. Por su parte, una muerte es prevenible cuando se puede evitar mediante intervenciones en salud pública, entendidas desde en un sentido amplio (Grupo B). Así, las muertes evitables corresponden a todas las definidas como tratables, prevenibles o mixtas (Grupo C) (10,11).

El objetivo de este trabajo fue estimar la magnitud de la proporción de muertes potencialmente evitables en niños menores de cinco años de Colombia, por departamentos y municipios, en el período 2000-2018, de manera global y por grupo de evitabilidad, utilizando una lista específica para Colombia, con el fin de identificar territorios con mayor peso de muertes evitables.

## MÉTODOS

Se llevó a cabo un estudio ecológico, longitudinal y en múltiples grupos. La unidad de análisis correspondió al municipio, para lo cual se utilizó la distribución político-administrativa de Colombia del año 2005. Los municipios creados con posterioridad fueron retornados a los municipios de segregación (Norosí, Guachené, San José de Uré y Tuchín). De la misma manera se procedió con Belén de Bajirá, debido a que, una vez resuelta la disputa territorial, regresó como corregimiento a Riosucio (Chocó). En este sentido, se analizaron 1 118 municipios en 19 años.

La fuente de información correspondió a las estadísticas vitales del Departamento Administrativo Nacional de Estadísticas (DANE). Se trabajó con los microdatos de las defunciones no fetales en niños menores de cinco años residentes colombianos, registrados entre los años 2000 y 2018; por otra parte, los datos geoespaciales se obtuvieron del Instituto Geográfico Agustín Codazzi.

Los microdatos fueron sometidos previamente al análisis de calidad. La preparación de la base de datos incluyó la imputación del municipio de residencia de la madre cuando el dato estuvo perdido (1,1%); este proceso se llevó a cabo de manera independiente para cada año. También se imputó por asignación proporcional, y teniendo en cuenta el sexo y la edad, la causa básica de muerte cuando (i) esta no era plausible en niños menores de cinco años; (ii) correspondía a un código que no aporta a la toma de decisiones en salud pública; o (iii) eran errores de digitación. En total, se recodificó la causa básica de muerte de 8,3% de los registros individuales.

Luego, se aplicó la lista de causas de muerte potencialmente evitables en niños menores de cinco años para Colombia, diferenciando las muertes evitables (tratables, prevenibles y mixtas) de las difícilmente evitables con el conocimiento y la tecnología actual.

Se calcularon proporciones de evitabilidad global y por grupo de evitabilidad (A, B y C) a nivel nacional, departamental y municipal. Se estimaron las desviaciones estándar inter e intradepartamentales y se compararon proporciones con la prueba c2 de independencia. Se calcularon variaciones porcentuales en el tiempo, y para cada uno de los niveles se aplicó la técnica multivariable de análisis de conglomerados de k-medianas con distancias euclidianas para discriminar cinco grupos. Para comparar los conglomerados se utilizó la prueba Kruskal Wallis.

Los resultados se presentan a través de gráficas de tendencias y mapas coropléticos. Para el procesamiento de la información se utilizó MS Excel, StataMP v14^®^ y Tableau^®^ Desktop v2020.1

Este proyecto fue avalado por el Comité de ética de la Investigación de la Facultad Nacional de Salud Pública (CI 341-2018) y catalogado como de riesgo mínimo. Al hacer uso del fuentes anonimizadas y agrupadas por territorios, se garantiza la protección de datos de los sujetos.

## RESULTADOS

### Muertes evitables de NM5 a nivel nacional

**FIGURA 1.**
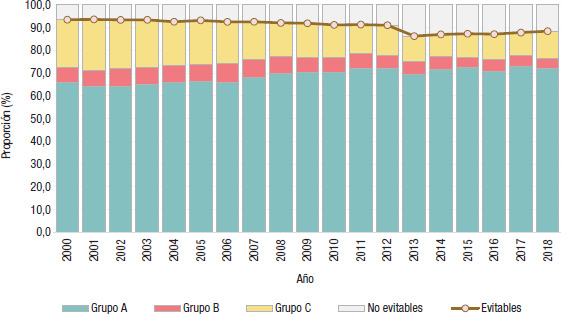
Distribución proporcional de las defunciones de niños menores de cinco años según el grupo de evitabilidad, Colombia (2000-2018)

Entre los años 2000 y 2018, se registraron en Colombia 228 942 defunciones de NM5, de las cuales 91,4% fueron clasificadas como potencialmente evitables (Figura 1), sin diferencias según el sexo (91,5% vs 91,4% para hombres y mujeres, respectivamente, c2 de independencia; *P* = 0,320). La proporción varió en el tiempo, con un leve descenso durante la serie, pasando de 93,5% a 88,5% (disminución relativa de 5,3%).

De acuerdo con los grupos de evitabilidad, en primer lugar, se ubicaron las muertes que pudieron evitarse por medio de atención médica oportuna y de calidad (Grupo A), de forma que 68,2% de todas las muertes registradas se clasificaron en este grupo. La participación porcentual de esta categoría aumentó en el tiempo, pasando de 65,9% en el año 2000 a 72,1% en 2018 (incremento relativo de 9,4%).

En segundo lugar, se posicionaron las muertes que pudieron evitarse a través de intervenciones mixtas (Grupo C). En este caso, 16,5% de las muertes pudieron evitarse tanto a través de intervenciones de promoción de la salud, prevención de la enfermedad y medidas de salud pública intersectoriales, como desde la adecuada atención médica con la aplicación de tratamientos bien conocidos en la actualidad.

Por otro lado, 6,8% de las muertes fueron clasificadas en el grupo de evitabilidad B, defunciones que pudieron prevenirse mediante intervenciones en salud pública. Esta categoría presentó la mayor disminución porcentual durante el período, pasando de 6,4% a 4,5% del total de las defunciones registradas (disminución de 29,4%).

Se puede señalar que 8,6% de las defunciones en niños menores de cinco años entre los años 2000 y 2018 eran difícilmente evitables con el conocimiento y avance tecnológico disponibles en el momento. La participación porcentual aumentó en el período, pasando de 6,5% a 11,5% entre los años 2000 y 2018, respectivamente.

***Fuente:*** elaboración propia.

### Muertes evitables de NM5 a nivel departamental

**FIGURA 2.**
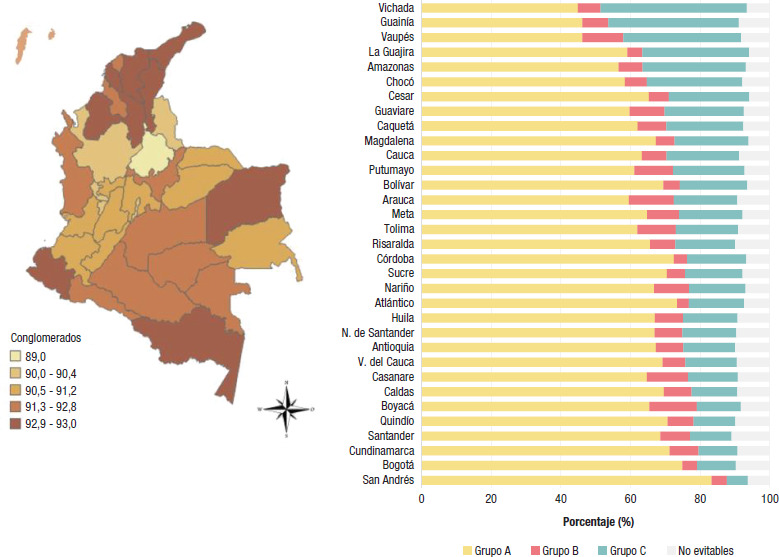
Proporción de muertes evitables según grupo de evitabilidad en niños menores de cinco años por departamento de residencia, Colombia (2000-2018)

De acuerdo con la distribución territorial, la proporción de evitabilidad entre los departamentos varió entre 89,0% y 94,1% (Figura 2), con diferencias estadísticas entre conglomerados (Kruskal Wallis; *P* = 0,0001). El primer conglomerado estuvo conformado exclusivamente por el departamento de Santander, donde se presentó la proporción de evitabilidad menos alta (89,0%); en contraste, el conglomerado con las proporciones más altas estuvo constituido por Cesar, La Guajira, Magdalena, San Andrés y Providencia, Bolívar, Vichada, Córdoba, Amazonas y Nariño, territorios donde las muertes de NM5 pudieron evitarse entre 93,0% y 94,1%.

Además, no solo se encontraron diferencias entre los departamentos, sino también dentro de ellos, especialmente en los municipios de Santander. La variación entre territorios indica un efecto grupal dado por el departamento de residencia (desviación estándar: 2,2 puntos), mientras que los municipios que conforman un departamento se alejan de la media grupal departamental en aproximadamente 7,1 puntos porcentuales.

De manera similar a la distribución nacional, la mayoría de las defunciones en todos los departamentos pudieron evitarse a través de la atención médica oportuna y de calidad (Grupo A), dicha proporción varió entre 44,9% y 83,2% de las muertes registradas (en Vichada y San Andrés y Providencia, respectivamente). Además, en algunos departamentos como Vichada, Guainía, Vaupés, La Guajira, Amazonas y Chocó también fueron importantes las proporciones de evitabilidad a través de intervenciones mixtas (Grupo C).

### Muertes evitables de NM5 a nivel municipal

**FIGURA 3.**
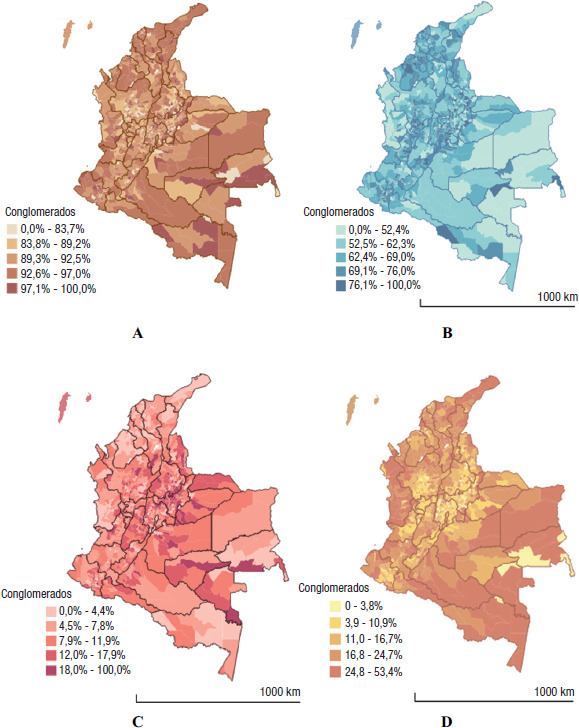
Proporción de muertes evitables total y según grupo de evitabilidad en niños menores de cinco años por municipio de residencia, Colombia (2000-2018). **A**, muertes evitables; **B**, muertes tratables a través de atención médica (Grupo A); **C**, muertes prevenibles a través de intervenciones en salud pública (Grupo B); y **D**, muertes evitables a través de acciones mixtas (Grupo C)

Como puede observarse en la figura 3a, existen diferencias entre los territorios en la proporción de muertes evitables entre los años 2000 y 2018 (Kruskal Wallis; *P* = 0,0001). La proporción de evitabilidad más baja se observó en el municipio del Palmar (Santander), donde 33,3% de las defunciones registradas se clasificaron como evitables, en contraste con 99 territorios donde 100% se clasificaron de la misma manera. Cabe resaltar que en los 19 años de análisis no se registraron defunciones de NM5 residentes de La Victoria (Amazonas), La Guadalupe (Guainía) o Morichal Nuevo (Guainía).

La proporción de las muertes tratables (Grupo A) a nivel municipal varió entre 20,0% (San Eduardo, Boyacá) y 100,0% (Jerusalén, Cundinamarca; La Uvita, Boyacá; y Papunaua, Vaupés) (Figura 3b). En contraste, en 67 municipios no se registraron muertes clasificadas en el Grupo B, mientras que en otros dos todas las muertes se clasificaron en ese grupo: Busbanzá, Boyacá y Pana Pana, Guainía (Figura 3c). Por último, las proporciones más altas de muertes clasificadas en el Grupo C (Figura 3d) se concentraron en los municipios de La Guajira, del Pacífico chocoano, de la Orinoquía y Amazonía, donde no solo es necesario mejorar el acceso a la atención médica, sino que también es determinante ejecutar estrategias de salud pública intersectoriales.

## DISCUSIÓN

La mayoría de las muertes de NM5 registradas en Colombia entre los años 2000 y 2018 fueron innecesariamente precoces y médicamente tratables. Solo una de cada diez muertes era difícilmente evitable según el conocimiento y la tecnología disponible al momento de la defunción. Si además se consideran las profundas diferencias observadas entre los territorios –sobre todo entre los municipios de residencia–, se advertirá que estas muertes pueden expresar inequidades en salud posiblemente originadas en las condiciones socioeconómicas, políticas y del sistema de salud en los territorios.

A continuación, se discuten cuatro asuntos: i) la dinámica temporal la tasa de MNM5 con relación al comportamiento de la fracción de evitabilidad en el país y en otros contextos; ii) la distribución de las muertes por grupos de evitabilidad y las diferencias de proporciones entre los territorios; iii) algunos aspectos metodológicos concernientes a esta investigación, y, por último, iv) las conclusiones y algunas recomendaciones en clave de política.

***Fuente:*** elaboración propia.

En cuanto a la dinámica temporal, se resalta que la mortalidad en la niñez ha disminuido de manera considerable en el país en términos relativos. Entre 1990 y 2018, la tasa pasó de 35 a 14 defunciones por cada 1 000 NV antes de cumplir los primeros cinco años de vida (1), lo cual constituye un avance importante en la supervivencia de la primera infancia colombiana. No obstante, en este estudio no se encontró una disminución similar en la proporción anual de muertes evitables en la niñez. Entre los años 2000 y 2018 el porcentaje de evitabilidad nacional pasó de 93,5% a 88,5%, de manera que en ese último año pudieron haberse evitado 7 755 muertes.

Las altas fracciones de evitabilidad potencial no son exclusivas para Colombia. De acuerdo con UNICEF y la Organización Mundial de la Salud (OMS), la mayoría de las muertes de NM5 en el mundo se pueden prevenir o tratar a través de intervenciones simples y bien conocidas, incluidas la inmunización, la nutrición adecuada, el agua potable y la atención oportuna en salud (7, 12).

Por otro lado, hay congruencia entre varias investigaciones de países del hemisferio sur en cuanto a los altos porcentajes de muertes evitables en NM5. Es el caso del estudio en dos distritos de Mali y tres distritos de Uganda entre 2011 y 2015, en el que 97% y 95% de las defunciones de NM5, respectivamente, presentó al menos un factor evitable (13).

Asimismo, algunas investigaciones en América Latina mostraron altas proporciones de muertes evitables en la niñez. En Brasil, más de la mitad de todas las muertes de NM5 han sido clasificadas como evitables por el Sistema Único de Salud, pasando de 75,7% en el año 2000 a 68,8% en 2013 (14). En Uruguay, 56,0% de las defunciones de niños menores de 14 años entre 2004 y 2006 en un hospital pediátrico eran evitables (15) y en Argentina (2017), a nivel nacional, 55,0% de las muertes neonatales y 68,0% de las posneonatales fueron potencialmente evitables (16). Estas cifras contrastan con las observadas en el Reino Unido, donde 35,0% de las muertes de niños y adolescentes fueron consideradas evitables (17). De esta manera, se evidencia una “brecha de evitabilidad” entre países europeos y los de América Latina, con características diferenciadoras, entre ellas, la concentración de la riqueza (18).

En cuanto a la distribución de las muertes según el grupo de evitabilidad, las defunciones de NM5 de Colombia entre los años 2000 y 2018 se clasificaron, principalmente, en el grupo de causas tratables (68,2%, Grupo A), –más las muertes evitables a través de acciones mixtas (Grupo C) 16,5%, en las que el acceso a la atención sanitaria también hubiera hecho la diferencia–. Vale la pena mencionar que el porcentaje de muertes tratables ha sido ampliamente utilizado para evaluar el desempeño de los sistemas nacionales de salud (10, 19-21).

***Fuente:*** elaboración propia.

Por su parte, las muertes que pudieron evitarse a través de acciones de salud pública (Grupo B) representaron la menor proporción de las defunciones (4,5%). En parte, este resultado puede relacionarse con los logros en la cobertura de vacunación para la prevención de eventos inmunoprevenibles (22); los niños han sido priorizados en el Plan Ampliado de Inmunizaciones (PAI) y los biológicos correspondientes en el esquema nacional de vacunación son gratuitos (23). De esta manera, en 2019 se obtuvo una cobertura de inmunización de 89,9% de la vacuna antituberculosa (BCG) y de 93,4% de la pentavalente (DTP-HiB-HB) en niños menores de un año; además, 94,3% de los niños de un año fueron vacunados con la triple viral (sarampión, rubéola y paperas [SRP]) (24). También se destaca que 86,1% de los NV en 2018 tuvo cuatro controles prenatales o más (24).

Como se evidencia en los resultados, existe alta heterogeneidad entre los municipios. Esta arista debe considerarse en nuevas investigaciones sobre desigualdades sociales en salud, en la medida que los resultados pueden relacionarse con el tamaño, las características y distribución poblacional, las condiciones socioeconómicas del municipio, la situación geográfica, la disponibilidad de servicios de salud a nivel territorial y las posibilidades y dificultades de acceso efectivo a los servicios de salud, entre otros.

En este sentido, Colombia se ha caracterizado por un desarrollo territorial desigual en términos socioeconómicos; las mediciones del índice de pobreza multidimensional evidencian brechas entre municipios, departamentos y regiones. El informe de 2018 del DANE destaca que la región Caribe y Pacífica (sin incluir el Valle del Cauca) tienen la mayor incidencia de pobreza multidimensional, 33,5% y 33,3% respectivamente; en contraste con Bogotá, Valle del Cauca y la región Oriental, donde existe una incidencia baja (4,3%, 13,6% y 16,4%, respectivamente) (25). Esta desigualdad regional se encontró también en esta investigación, razón por la cual resultaría pertinente analizar en conjunto las características de contexto a nivel territorial y las diferencias en la mortalidad potencialmente evitable de NM5 en los departamentos y municipios colombianos.

Entre los aspectos metodológicos concernientes a este estudio, se debe destacar la utilización de la distribución político-administrativa de Colombia vigente para el año 2005; los municipios creados posteriormente fueron reintegrados al municipio segregante. Esta consideración es importante en la medida que los territorios tienen un comportamiento dinámico, y porque el lugar incide sobre el proceso salud-enfermedad de las personas que lo habitan (26), en tanto que este puede condicionar su supervivencia, la calidad de vida, el acceso a recursos, las prácticas culturales y su salud en general (27).

Además, el objetivo de la investigación fue evidenciar el comportamiento de la evitabilidad de las muertes observadas en NM5; sin embargo, no se consideraron los denominadores poblacionales –como los NV– para hacer comparaciones en la magnitud del evento entre poblaciones. En este caso, se estableció la proporción de muertes evitables como fracción del total de las defunciones registradas y no se estimó el riesgo de que un niño nacido en determinado territorio y año muriera por una causa potencialmente evitable antes de cumplir los primeros cinco años. Dicho análisis debe realizarse a futuro para nutrir y complementar estos resultados.

Este estudio presenta algunas limitaciones; entre ellas, las propias a la utilización de fuentes secundarias de información. Llamó la atención que en tres territorios no municipalizados –La Victoria, La Guadalupe y Morichal Nuevo– no se notificaron defunciones de NM5 en los 19 años de estudio. Esta característica se debe más a problemas de subregistro de las estadísticas vitales que a la ausencia real de muertes en los lugares; el DANE estimó las tasas de mortalidad infantil y en la niñez para dichos territorios y el resultado fue superior a la media nacional (28). Así, se deben asumir los problemas de cobertura y el nivel de subregistro de la fuente de información.

Por otra parte, debido a las diferencias metodológicas y a la utilización de distintos criterios para valorar la evitabilidad potencial de las causas de muerte, las comparaciones con los resultados de estudios internacionales deben realizarse con cautela.

A pesar de dichas limitaciones, se resalta la utilización del diseño ecológico para abordar este tema y reconocer la fracción de evitabilidad en las muertes de NM5 en los territorios colombianos en el tiempo, lo cual ofrece conocimiento pertinente para determinar objetivos de prevención, prioridades y estrategias para los tomadores de decisiones en el país.

### Conclusiones

El número de muertes potencialmente evitables sobrepasaron en gran cantidad las muertes no evitables de NM5 de Colombia, sin diferencias según el sexo. Las defunciones pudieron haberse evitado, principalmente, a través del acceso efectivo y oportuno a la atención sanitaria. Además, las diferencias en las proporciones de evitabilidad según el municipio de residencia sugieren la existencia de inequidades en salud posiblemente originadas por inequidades estructurales entre los territorios que deben ser subsanadas.

Debido a que la mortalidad infantil la niñez es muy sensible a las condiciones de vida y al desarrollo de la sociedad, es bastante probable que las brechas territoriales puedan estrecharse o eliminarse a través de la mejora de las condiciones de vida de la población pobre y vulnerada garantizando, entre otras, la cobertura de agua potable, el acceso a alimentos suficientes en cantidad y calidad, el acceso de calidad a la educación (especialmente de las mujeres), el saneamiento básico y el empleo digno. También se precisa la garantía y el goce efectivo del derecho fundamental a la salud y la transición hacia un sistema de salud que tenga en cuenta las diferencias entre los territorios, con oferta suficiente de servicios sanitarios en todo el país. Para lograr estas condiciones, es necesario el trabajo conjunto de los gobiernos nacional, departamentales y municipales, instituciones oficiales, organizaciones de la sociedad civil y del compromiso de la sociedad en general para garantizar el goce efectivo de los derechos fundamentales de los niños, entre ellos a la salud y a la seguridad social, a la integridad física y a la vida.

## Declaración.

Las opiniones expresadas en este manuscrito son únicamente responsabilidad de los autores y no reflejan necesariamente los de la *Revista Panamericana de Salud Pública* o la Organización Panamericana de la Salud.
